# *Strong*-LAMP Assay Based on a *Strongyloides* spp.-Derived Partial Sequence in the 18S rRNA as Potential Biomarker for Strongyloidiasis Diagnosis in Human Urine Samples

**DOI:** 10.1155/2020/5265198

**Published:** 2020-05-31

**Authors:** Pedro Fernández-Soto, Carmen T. Celis-Giraldo, Coralina Collar-Fernández, Óscar Gorgojo, Milena Camargo, José Muñoz, Joaquín Salas-Coronas, Manuel A. Patarroyo, Antonio Muro

**Affiliations:** ^1^Infectious and Tropical Diseases Research Group (e-INTRO), Biomedical Research Institute of Salamanca-Research Centre for Tropical Diseases at the University of Salamanca (IBSAL-CIETUS), Faculty of Pharmacy, University of Salamanca, Salamanca 37007, Spain; ^2^Animal Science Faculty, Universidad de Ciencias Aplicadas y Ambientales (U.D.C.A), Bogotá 111166, Colombia; ^3^Molecular Biology and Immunology Department, Fundación Instituto de Inmunología de Colombia (FIDIC), Bogotá 111321, Colombia; ^4^PhD Programme in Biomedical and Biological Sciences, School of Medicine and Health Sciences, Universidad del Rosario, Bogotá 112111, Colombia; ^5^ISGlobal, Barcelona Ctr. Int. Health Res. (CRESIB), Hospital Clínic-Universitat de Barcelona, Barcelona 08036, Spain; ^6^Unidad de Medicina Tropical, Hospital de Poniente, El Ejido 04700, Almería, Spain; ^7^Basic Sciences Department, School of Medicine and Health Sciences, Universidad del Rosario, Bogotá 112111, Colombia

## Abstract

Human strongyloidiasis a soil-transmitted infection caused by *Strongyloides stercoralis* is one of the most neglected amongst the so-called Neglected Tropical Diseases (NTDs). *S. stercoralis* is a nematode, which is distributed worldwide; it has been estimated that it could affect millions of people, mainly in tropical and subtropical endemic regions. The difficulties of diagnosis lead to infection rates being underreported. Asymptomatic patients have chronic infections that can lead to severe hyperinfection syndrome or disseminated strongyloidiasis in immunocompromised patients. Strongyloidiasis can easily be misdiagnosed because conventional faecal-based techniques lack of sensitivity for the morphological identification of infective larvae in faeces. None of the currently used molecular methods have used urine samples as an alternative to faecal samples for diagnosing strongyloidiasis. This study was thus aimed at comparing, for the first time, the use of a new loop-mediated isothermal amplification (LAMP) molecular assay (*Strong-*LAMP) to traditional methods on patients' urine samples. Twenty-four urine samples were taken from patients included in a study involving two Spanish hospitals for strongyloidiasis screening using parasitological and serological tests. *Strongyloides* larvae were found in 11 patients' faecal samples, thereby ascertaining that they had the disease. Other patients had high antibody titres but no larvae were found in their faeces. All urine samples were analysed by PCR and *Strong-*LAMP assay. No amplification occurred when using PCR. *Strong-*LAMP led to detecting *S. stercoralis* DNA in urine samples from patients having previously confirmed strongyloidiasis by parasitological tests and/or a suspicion of being infected by serological ones. The *Strong*-LAMP assay is a useful molecular tool for research regarding strongyloidiasis in human urine samples. After further validation, the *Strong*-LAMP assay could also be used for complementary and effective diagnosis of strongyloidiasis in a clinical setting.

## 1. Introduction

Strongyloidiasis is an infection caused by the parasitic nematodes from the genus *Strongyloides*: *S. stercoralis* and to a lesser extent *Strongyloides fuelleborni.* Originally known as “anguilulosis” or “Cochinchina diarrhoea”, the World Health Organisation (WHO) now considers it a neglected tropical disease (NTD) [1, 2]. *S. stercoralis* has a cosmopolitan distribution in tropical and subtropical regions [3]. It can also be found in temperate areas, such as the Mediterranean region, southern USA, and Japan. Regarding *S. fuelleborni*, although primarily affecting nonhuman primates, human cases have also been described in Africa and Southeast Asia, mainly in Papua New Guinea [4, 5].

Strongyloidiasis worldwide has currently been calculated as ranging from 30-100 million infected people, mainly in low-income countries and those having poor sanitary conditions [6, 7]. Such variation regarding its estimation is largely due to its asymptomatic clinical picture, the tremendous difficulties regarding its diagnosis, and the affected people's lack of access to a health system. The disease's prevalence is considered to be greatly underestimated [8]. *S. stercoralis* is an autochthonous parasite in Spain all along its Mediterranean coastline, particularly in La Safor region within the province of Valencia, Spain, where it reaches 12.4% in high-risk groups related to agricultural work [6, 9]; cases have also been reported on the banks of the Ebro river [10]. Most European cases have been concerned with parasitosis imported by immigrants from strongyloidiasis-endemic areas, to a lesser extent, cases of travellers visiting such areas [11, 12] .

Strongyloidiasis clinical manifestations depend on parasite development and invasion stage, its self-infection capability, and a patient's immunological state. This may appear as an acute infection and chronic infection and produce a hyperinfection syndrome and/or a disseminated infection. Acute strongyloidiasis is not common and usually appears in travellers returning from a highly-endemic area suffering from pruritic dermatitis (due to the larvae penetrating the skin), pneumonitis accompanied by cough and expectoration (when the larvae enter the lungs), and fever. The parasites produce gastrointestinal pain accompanied by diarrhoea, nausea, and, occasionally, vomiting when they reach the intestines. Chronic (or low intensity) strongyloidiasis is usually asymptomatic, although it can have slight to moderate symptomatology, accompanied by gastrointestinal, pulmonary and cutaneous manifestations, and eosinophilia (in 75% of patients) [13].

It can produce the hyperinfection syndrome in immunosuppressed individuals when the larvae migrate, accompanied by more severe intestinal and pulmonary manifestations, fever, weakness, and a greater amount of larvae in faeces and sputum. Immunosuppressive treatments involving corticosteroids, solid or haematopoietic organ transplants, cancer, and HTLV-1 infection are considered the most important associated risk factors [4], along with malnutrition and associated infections in areas having high endemicity [14]. Anti-TNF therapies (stand alone or in combination with glucocorticoids) have favoured the development of clinical pictures and hyperinfections as they affect Th2 cells' immune response [15, 16].

The larvae can cross the blood-brain barrier, producing encephalitis and up to 87% mortality rates. The treatment usually used for strongyloidiasis is no longer effective at this point [17]; screening individuals suspected of having strongyloidiasis before immunosuppressive treatment is thus essential [4]. Ivermectin has been seen to be the most therapeutically effective drug used in control strategy; it continues being the drug of first choice regarding other options such as albendazole, thiabendazole, or mebendazole which are less effective and less safe [6, 16, 18, 19].

However, diagnosis is undoubtedly the main problem regarding strongyloidiasis due to little knowledge being available concerning the disease, its effects in nonendemic areas, current diagnostic techniques having little sensitivity and specificity, the parasitological methods requiring specialised personnel, and centres and no gold standard for diagnosis. This means that the case definition and the possible validation of new diagnostic methods are enormously hampered [20].

Current parasitological and immunological *S. stercoralis* diagnostic methods are thus being complemented by molecular methods [17, 21, 22]. Different approaches to the molecular detection of *S. stercoralis* in faecal samples have been developed from the description of the *Strongyloides* spp. 18S ribosomal subunit sequence, using polymerase chain reaction (PCR), both simple and nested techniques, and real time-PCR (RT-PCR) [8, 23]. Another recent molecular alternative method for diagnosing strongyloidiasis in patients' faecal samples [24] is the loop-mediated isothermal amplification (LAMP) of nucleic acids which has numerous advantages over other more complex molecular diagnosis techniques [25, 26].

LAMP is currently considered a technique having great potential for use in field conditions, mainly in endemic areas, as a future, highly effective, point-of-care testing method [27]. Fernández-Soto and colleagues [28] have developed a new LAMP method called *Strong*-LAMP for the molecular detection of *Strongyloides* spp. in urine and faecal samples in a murine model. It has also been used for analysing stool samples from patients previously diagnosed by parasitological and molecular (RT-PCR) methods, thereby making it a highly efficient diagnosis technique [28]. In this work, the *Strong*-LAMP is used for the first time in human urine samples from patients attending at two hospitals in Spain and compared with parasitological and serological methods.

## 2. Materials and Methods

### 2.1. Obtaining the Samples

The urine samples used in this study were obtained from patients (mostly immigrants) attending the Tropical Medicine Unit in Hospital Clínic de Barcelona (Barcelona, Spain) and Hospital de Poniente (El Ejido Almería, Spain) as part of a strongyloidiasis diagnosis screening study. The inclusion criteria were being a patient having simultaneous blood, faeces, and urine samples; having a positive parasitological and/or serological diagnosis for Strongyloides who had been recruited for a multicentre study for strongyloidiasis diagnosis; a convenience sampling was thus performed, considering the abovementioned criteria. Each individual's clinical picture was recorded. All the patients included in the study were first analysed by *S. stercoralis* serology using a Microwell ELISA kit (IVD Research, Inc., Carlsbad, CA) (https://ivdresearch.com/elisa/strongyloides-serum-antibody-detection-microwell-elisa/) and coproparasitological analysis performed on only one sample (Ritchie technique and agar-plate culture for patients attending the Hospital de Poniente and just agar-plate culture for the Hospital Clínic de Barcelona patients) for detecting *S. stercoralis*. Samples whose optical density (OD) values were ≥1 in the IVD-ELISA test were considered positive (according to the manufacturers' specifications). The study involved 24 urine samples: 16 from Hospital Clínic (Barcelona) and 8 from Hospital de Poniente (Almería). All samples were sent to CIETUS in 10 mL tubes and stored frozen until use.

### 2.2. Ethical Considerations

Both hospitals' Ethics Committees approved the study; the urine samples were obtained after the patients had signed informed consent forms regarding their analysis.

### 2.3. Obtaining and Preparing the DNA Samples

#### 2.3.1. Obtaining and Preparing *Strongyloides venezuelensis* DNA

DNA from *S. venezuelensis* infective filiform larvae (L3) was used as amplification positive control for PCR and LAMP reactions; their biological cycle is routinely maintained in experimentally-infected Wistar rats in the CIETUS, Universidad de Salamanca. A NucleoSpin Tissue kit (Machery-Nagel) was used for extracting DNA from the larvae, following the manufacturers' instructions. The DNA concentration was measured on a NanoDrop spectrophotometer (ND-1000) and adjusted to the final 5 ng/*μ*L concentration. This DNA (2 *μ*L) was then used as positive control for all subsequent PCR and LAMP reactions.

#### 2.3.2. Obtaining and Preparing DNA from Patients' Urine

This involved taking 2 mL aliquots of patients' urine from the unfrozen vials; the rest remained frozen at -20°C. The vials to be analysed were spun at 4,000 rpm for 15 min to obtain sediment for extracting the DNA using the i-genomic Urine DNA Extraction Mini Kit (Intron Biotechnology), following the manufacturers' instructions. A NanoDrop spectrophotometer (ND-1000) was used for measuring the DNA concentration from each urine sample (100 *μ*L elution volume); they were then labelled and stored until use at -20°C in two vials (50 *μ*L in each).

### 2.4. Amplification Target and Specific Primers for LAMP Amplifying *Strongyloides* spp. DNA

The *Strong*-LAMP method previously validated and developed at CIETUS was used for *Strongyloides* spp. DNA amplification [17]. Briefly, the selected amplification target was a *Strongyloides venezuelensis* partial 18S rRNA gene (GenBank Accession number: AJ417026.1) 329 base pair (bp) sequence; Primer Explorer V.4 software (https://primerexplorer.jp/e/) was used for designing a set of four primers (F3, B3, FIP, and BIP) on this sequence (Table 1 shows the selected primer sequences). A Fisher Scientific synthesis kit was used, and purified products were suspended in ultrapure water at 100 pmol/*μ*L final concentration.

### 2.5. Molecular Analysis of Patients' Urine Samples

#### 2.5.1. PCR Analysis of F3 and B3 External Primers

The urine samples were analysed by PCR using F3 and B3 external primers from the set of 4 primers for the LAMP assay; correct PCR functioning had already been verified with *S. venezuelensis* DNA. A touchdown PCR (TD-PCR) was carried out consisting of decreasing the annealing temperature by one degree per each amplification cycle for guaranteeing a suitable range of annealing temperatures for correct target sequence amplification [29]; briefly, TD-PCR was performed as follows: 94°C for 60 seconds and a touchdown program involving 18 cycles; Table 2 shows the reaction mixture and amplification conditions used in the TD-PCR. A 96-well thermal cycler (Gradient Mastercycler, Eppendorf) was used for all PCR reactions.

#### 2.5.2. LAMP Analysis of Patients' Urine Samples

All the urine samples were analysed by the *Strong-*LAMP previously described by Fernández-Soto et al. [28].  Table 3 describes the reaction mixture used. All reactions were incubated for 60 min at 63°C in a heating block (K Dry-Bath) plus 10 min at 80°C for deactivating the enzyme and stopping the reaction; 2 *μ*L DNA from each urine sample were used for amplification; *S. venezuelensis* DNA (2 *μ*L) and ultrapure water instead of DNA (2 *μ*L) were used as positive and negative controls, respectively.

#### 2.5.3. Detecting the Amplification Products

*(1) PCR*. PCR amplification products were detected on 1.5% agarose gels (100 mL 0.5X TBE, 1.5 g agarose), stained with ethidium bromide at 60 V for 20 min and then at 90-100 V for one hour. The gels were visualised and photographed using an ultraviolet imaging system (UVITEC Gel Documentation System, Cambridge, UK).

*(2) Strong-LAMP*. LAMP amplification products were visually detected by observing white turbidity at the bottom of the reaction tube and by colorimetric change on adding 2 *μ*L SYBR Green I fluorescent dye (Invitrogen) (1 : 10; 10,000X) to each reaction tube. Green indicated a positive result and orange a negative one (i.e., maintaining the dye's original colour). Colorimetric results were verified by 1.5% agarose electrophoresis for observing the characteristic pattern of bands which appears in positive LAMP results. The gels were then photographed and the images saved in digital format for editing.

### 2.6. Statistical Analysis

Stata MP 14.0 statistical software was used for analysing the data. A descriptive analysis was made; quantitative variables are shown as Medians or Means with their corresponding dispersion measures (Interquartile range-IQR or Standard Deviation-SD); 5% significance confidence intervals were calculated for each test. Agreement between serological and coproparasitological screening tests with *Strong*-LAMP results in this study was quantified and analysed using the kappa coefficient (*κ*), interpreted as: <0.00 poor, 0 ≤ *κ* ≤ 0.2 slight, 0.21 ≤ *κ* ≤ 0.40 fair, 0.41 ≤ *κ* ≤ 0.60 moderate, 0.61 ≤ *κ* ≤ 0.80 substantial, and >0.80 almost perfect [30].

## 3. Results

### 3.1. Serological and Parasitological Data

The study results showed that 54% of the samples came from Latin-American patients (mainly from Bolivia (9/24) and the remainder from Africa (specifically Gambia (4/24) and Guinea-Bissau (2/24)). The patients' epidemiological data stated that 62.5% (15/24) were male, most being aged from 30 to 60 years old (75%), with a mean = 41.1 (SD = 12.6). Regarding their clinical pictures, 50% (12/24) of the target population had symptoms such as abdominal pain, diarrhoea, urticaria, and pruritus. Eosinophilia was observed in 45.8% (11/24) of the population, with a median = 870 (IQR = 680 − 1900), accompanied by high IgE levels in 52.15% of them (12/23), with a median = 1320.5 (IQR = 542 − 2438) (Table 4). Strongyloidiasis diagnosis test revealed that 87.5% of the study population had ≥1 titres for IVD-ELISA with a median = 2.22 (IQR = 1.71 − 4.52). However, 37.5% (0.19-0.614 95% CI) of the population was positive by coproparasitological analysis by agar culture plate method (individual results in Supplementary material Tables 1 and 2. Once diagnosed, 95.8% (23/24) of the individuals were treated with ivermectin; all but one patient were followed-up.

### 3.2. Human Urine Samples Analysed by TD-PCR F3-B3 Assay

**Figure 1:**
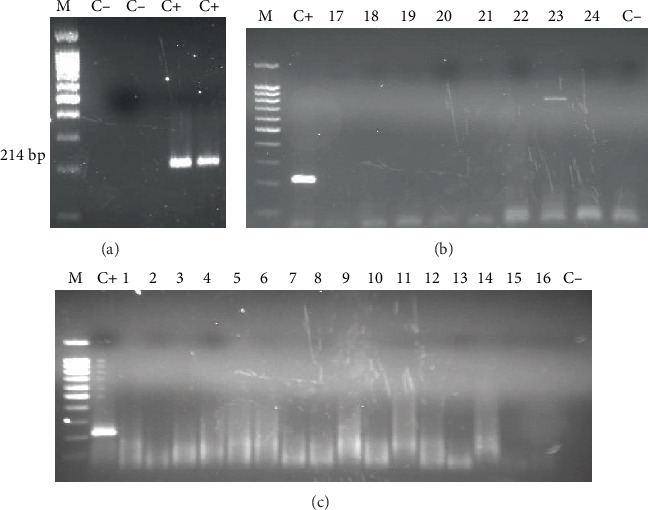
TD-PCR analysis of patients' urine samples. (a) PCR for verifying F3 and B3 primer functioning for amplifying the 214 bp fragment. (b) Samples from the Hospital de Poniente (El Ejido, Almería). (c) Samples from the Hospital Clínic de Barcelona. M: molecular weight marker (DNA Ladder 100 bp PLUS BLUE); C+: *Strongyloides venezuelensis* DNA (positive control); C−: ultrapure water, no DNA (negative control).

Figure 1 shows the TD-PCR amplification results; there was no amplification in any of the samples analysed.

### 3.3. *Strong*-LAMP Analysis of Patients' Urine Samples

**Figure 2:**
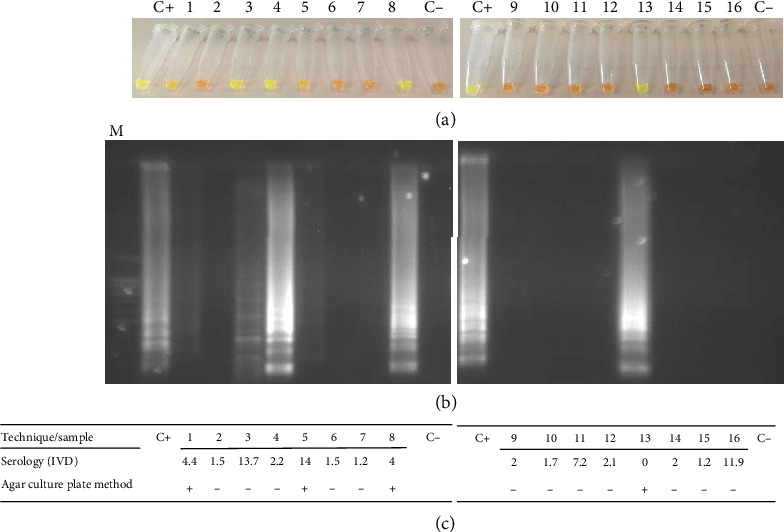
*Strong*-LAMP analysis of urine samples from patients attending Hospital Clínic de Barcelona (Barcelona). (a) Colorimetric results from adding SYBR Green I. (b) Agarose gel electrophoresis results. (c) Serological (IVD index, OD values) and agar plate culture results. M: molecular weight marker (DNA Ladder 100 bp PLUS BLUE); C+: *Strongyloides venezuelensis* DNA (positive control); C-: ultrapure water, no DNA (negative control).

**Figure 3:**
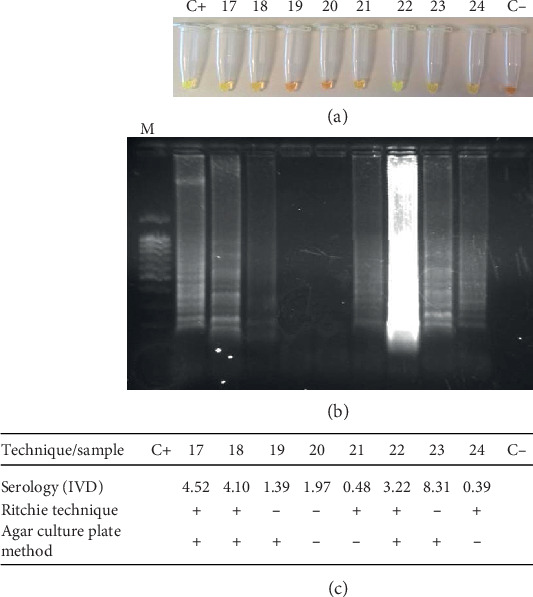
*Strong*-LAMP analysis of urine samples from patients attending Hospital de Poniente (El Ejido, Almería). (a) Colorimetric results by adding SYBR Green I. (b) Agarose gel electrophoresis results. (c) Serological (IVD index, OD values), Ritchie technique and agar plate culture results. M: molecular weight marker (DNA Ladder 100 bp PLUS BLUE); C+: *Strongyloides venezuelensis* DNA (positive control); C-: ultrapure water, no DNA (negative control).

It was found that 50% of the individuals (0.25-0.67 95% CI) were positive according to test parameters.  Figure 2 shows *Strong*-LAMP results from analysing the patients' urine samples from the Hospital Clínic de Barcelona and Figure 3 from the Hospital de Poniente; results from other tests are also shown.

### 3.4. Agreement between Screening Analysis and *Strong*-LAMP

**Figure 4:**
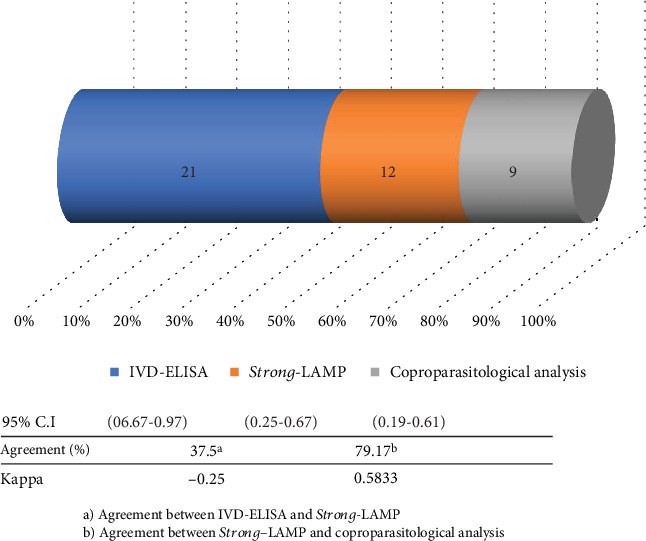
Agreement between *Strong*-LAMP, IVD detection techniques, and coproparasitological analysis in the studied samples with strongyloidiasis.

*Strong*-LAMP results were compared to the IVD test results and then to the coproparasitological analysis results. The numbers in the figure represent the total of samples proving positive in each test analysed. The scale on the lower axis indicates the percentage in relation to the total of positive individuals (Figure 4); 37.5% agreement was observed and poor correlation according to *κ* value for IVD and *Strong*-LAMP analysis (*κ* = −0.25; *p* = 0.968). Such data differed from that observed regarding coprology and *Strong*-LAMP results where agreement was 79.17%. The *κ* value gave moderate correlation (*κ* = 0.5833; *p* = 0.0016), according to the classification described above.

## 4. Discussion

One of the main problems regarding strongyloidiasis continues being the lack of a true gold standard for diagnosing the disease. Even though parasitological methods continue being considered “clinical or certainty diagnosis” (i.e., they enable visualising the larvae in faecal samples), they involve great disadvantages, mainly regarding their sensitivity [6, 21].

Serological methods also have problems regarding sensitivity (especially during the disease's acute phase) and specificity due to often producing crossed reactions concerning a diagnosis of other nematodes. PCR-based molecular methods and variants (e.g., RT-PCR) have been advanced during recent years as the most suitable ones for diagnosing strongyloidiasis, even though requiring complex infrastructure and specialised equipment and personnel which are not available in routine practice and are expensive and very difficult to perform in field conditions in strongyloidiasis-endemic areas [17, 31].

The lack of a suitable and standardised diagnosis method has led to underestimating the disease's real prevalence. Although migrants were screened in this study, it was interesting that most individuals came from Latin America, mainly from Bolivia. Studies have been inconsistent when reporting the disease's prevalence in Latin America; values have been higher than 20% in Argentina, Ecuador, Venezuela, Perú, and Brazil [32] whilst a study by Gétaz [33] in Bolivia estimated prevalence at 20.0%. Eosinophilia being found in 45.8% (11/24) of the target population accompanied by increased IgE production in 9 patients is parameters which have been reported in strongyloidiasis cases [34]. However, individuals suffering eosinophilia cannot always be identified as this depends on the parasite's cycle and concomitant infections [35]. Regarding IgE levels, normal levels have been reported in individuals infected by *Strongyloides stercoralis* who also proved positive for human T-cell lymphotropic virus type 1 (HTLV-1) [36].

Difficulties regarding diagnosis are especially important regarding immunocompromised people or immunosuppression candidates (e.g., in transplants) who could develop hyperinfection leading to a fatal outcome for the patient [4]. A study by Luvira et al. [37], involving patients who were immunocompromised due to different causes, analysed factors such as age, gender, and level of immunocompromise and reported that they were not associated with strongyloidiasis; prevalence ranged from 5%-7% and coproparasitological analysis in agar was the most sensitive technique in this study [37]. New sensitive, specific, technically easy, and economically affordable diagnosis methods must thus be developed; they must also become incorporated into routine clinical practice as a screening method for strongyloidiasis in patients who are candidates for immunosuppressive treatment.

However, most molecular diagnosis methods for detecting *S. stercoralis* have been based on using faecal or soil samples in the search for larvae. Faecal samples involve problems associated with collecting (serial, at least 3 samples), processing (usually requiring them to be concentrated), and storing them. Urine analysis is an alternative for making a diagnosis [20, 28, 38, 39]. A new molecular diagnosis method such as LAMP for use on urine samples thus provides an interesting approach, combining this technique's advantages over other molecular methods (i.e., PCR) and the advantages of analysing urine samples. Previous studies have evaluated the parasite's presence in urine; a study by Lodh et al. [40] detected *S. stercoralis-*derived DNA by molecular methods, Formenti et al. [41] evaluated parasite diagnosis by conventional and molecular methods such as q-PCR analysing urine samples and faecal material; they found that qPCR urine analysis sensitivity was just 17% compared to faecal material analysis having 63% sensitivity [41].

In a previous work, *Strong*-LAMP adaption of the LAMP method for detecting *Strongyloides venezuelensis* has proved effective regarding faecal and urine samples in an experimental infection model involving rodents and also regarding faecal samples from patients having a confirmed *S. stercoralis* infection [28]. The present work has evaluated, for the first time, the *Strong*-LAMP method as a molecular method for detecting *S. stercoralis* in patients' urine samples.

PCR was initially used with F3 and B3 external primers (even though none of the samples became amplified) for verifying whether PCR could be used as a molecular method for analysing urine samples. Single PCR has been used in other studies providing good results for detecting *S. stercoralis* DNA in faecal samples and has been prosed as a complementary molecular method to larval concentration in faecal samples before DNA extraction [42]. PCR F3-B3 (with 0.01 ng *Strongyloides* spp. DNA detection limit) has been effective in detecting *S. venezuelensis* in faecal samples in an experimental infection model [28].

However, positive amplification results have not been obtained when using PCR F3-B3 on patients' urine samples, meaning that it cannot be proposed as a complementary molecular method for a definitive diagnosis of strongyloidiasis. Nevertheless, using the *Strong*-LAMP method with samples has led to positive visible amplification results concerning colorimetric change and resolving reaction products on agarose gels.

Eleven of the 24 urine samples analysed had parasitologically positive results for *S. stercoralis* by at least one of the coproparasitological diagnosis methods (i.e., Ritchie technique and/or agar plate culture), and 45.83% of the samples were positive by *Strong*-LAMP. Furthermore, 16% of the samples turned a less pronounced green (indicating amplification) than the rest of the positive samples, and a pattern of bands was obtained which was dimmer on agarose gel. The high protein concentration in these samples could have limited amplification; the 260/280 ratio for absorbance regarding DNA extracted from the samples (often less than 1, most being around 0.5) was well below the expected value for pure nucleic acids (between 1.8 and 2.0; ≥1.8 for DNA), thereby limiting analytical purposes. Such high protein concentration would have been the main cause of inhibiting PCR analysing the samples.

*Strong*-LAMP amplification of two urine samples (I.D: 3 and 4) was obtained in patients having negative faecal sample results by agar plate culture. Given poor sensitivity for coproparasitological technique when analysing a single sample of a patient's faeces, both samples could have proved positive if a serial analysis had been performed (at least three samples from the same patient, taken on alternating days) for visualising *S. stercoralis* larvae [21].

Regarding serological analysis results, most samples had a serological IVD index greater than 1 OD, considered the cut-off limit for considering a result positive by this technique [43]. For example, the sample 3 had a very high IVD index (13.7 OD), indicating anti-*S. stercoralis* antibodies. It is worth noting that sample 13 which was positive by both agar plate culture and *Strong*-LAMP had an IVD serological index considered negative (0.005 OD). It has been noted that false negatives sometimes appear when using the IVD serological test [43]. Only one individual who was HIV positive (stage A2) proved positive in all tests carried out in this study. The tests are more efficient for detecting the parasite in such individuals since they may have a hyperinfection syndrome, as observed in a case report by Grossi et al. [44] describing a HIV-positive male having peripheral T-cell lymphoma who developed hyperinfection syndrome and was successfully treated with subcutaneous ivermectin [44]. A study in Brazil by Marchi-Blatt and Aparecida-Cantos [45] compared *Strongyloides stercoralis* diagnosis techniques on HIV positive and negative patients; it found that HIV positive patients had higher *S. stercoralis* infection frequency (odds ratio = 5.687) [45]. A study regarding urine analysis in Ethiopia by Hailegebriel et al. [46] demonstrated that agreement between coproparasitological analysis and *Strong*-LAMP for urine samples in this study was 78%; it should thus be worthwhile evaluating the test's efficiency in a broader population.

Despite the fact that PCR using external primers F3-B3 and the *Strong*-LAMP method has been shown to have the same limit for detecting *Strongyloides* spp. DNA (0.01 ng) [28], the results obtained from analysing the urine samples were consistent with the LAMP method having greater tolerance as described by other authors concerning possible inhibitors in biological samples analysed regarding PCR [47].

Although more studies are needed which would involve increasing the amount of urine samples to be analysed, the present work's results suggest that the *Strong*-LAMP method and those obtained in faeces [28] have demonstrated that the technique could be used as a complementary molecular tool when diagnosing strongyloidiasis. It could be used as a routine molecular method as it has certain advantages over PCR, i.e., it is cheaper as samples are amplified at constant temperature (59-66°C) making equipment such as water baths and thermal blocks sufficient, it can detect small concentrations of DNA in samples and is highly specific making it a simple technique to use [48–50].

## 5. Conclusions

The *Strong*-LAMP molecular diagnosis method was used for the first time for detecting *Strongyloides* spp. DNA in patients' urine samples. The *Strong*-LAMP method proved effective in detecting *S. stercoralis* DNA in the urine samples of patients with confirmed strongyloidiasis and/or the serological suspicion of infection by the parasite. The *Strong*-LAMP method could be used on urine samples as a complementary method for detecting *S. stercoralis* infection.

## Figures and Tables

**Table 1:** Nucleotide sequences from a set of primers selected from the 329 bp sequence (GenBank Acc. num.: AJ417026.1) for LAMP amplification of *Strongyloides* spp. DNA [28].

Primer	Length (bp)	Sequence (5′-3′)
F3	21	ACACGCTTTTTATACCACATT
B3	18	GTGGAGCCGTTTATCAGG
FIP	49	ACCAGATACACATACGGTATGTTTTGGATTTGATGAAACCATTTTTTCG
BIP	43	ATCAACTTTCGATGGTAGGGTATTGCCTATCCGGAGTCGAACC

**(a):** 

Components	Volume (*μ*L)
H_2_O	17.1
10x buffer	2.5
MgCl2 (25 mM)	1.5
dNTPs (2.5 mM)	0.5
F3 (pmol)	0.5
B3 (pmol)	0.5
Taq polymerase (2 U)	0.4
Template DNA	2
Total	25

**(b):** 

Temperature (°C)	Time (sec)	Cycle
94	60	X 1
94	20	X 2
57-52	20	
72	30	
94	60	X 15
51	20	
72	30	
72	60	X 1

**Table 3:** Reaction mixture used in *Strong*-LAMP assays. *Bst* polymerase, buffer, and MgSO_4_ were supplied by New England Biolabs, betaine by SIGMA, and the dNTPs by Intron.

Component	Volume (*μ*L)
H_2_O	7.7
Betaine (1 M)	5
MgSO_4_	1.5
dNTPs (2.5 mM)	3.5
10x buffer	2.5
FIP (40 pmol/*μ*L)	0.4
BIP (40 pmol/*μ*L)	0.4
F3 (5 pmol/*μ*L)	0.5
B3 (5 pmol/*μ*L)	0.5
Bst polymerase 2.0	1
DNA	2
Total	25

**(a):** 

Epidemiological characteristic	Total (*n* (%))
Origin	
Bolivia	9 (37.5)
Gambia	4 (16.6)
Ecuador	2 (8.3)
Guinea Bissau	2 (8.3)
Spain	2 (8.3)
Other^1^	5 (20.83)
Gender	
Female	9 (37.5)
Male	15 (62.5)
Age (years)	
<30	4 (16.6)
30-60	18 (75)
>60	2 (8.3)
Clinical status	
Asymptomatic	12 (50)
Symptomatic	12 (50)

**(b):** 

Laboratory test	*n*	Median	IQR
Haematological result			
Eosinophils	23	400	(100-870)
IgE	22	358	(72-1653)

^1^Only one person by country (Colombia, Cuba, Mali, Nigeria, and Portugal). ^2^Optical density (OD) according to the maker's specifications.

## Data Availability

All data used to support the findings of this study are included in the manuscript and figures.

## Supplementary Materials

Supplementary Materials Table 1. Individual patients results from the Hospital Clínic de Barcelona, Barcelona, Spain. Table 2. Individual patients results from Hospital de Poniente, El Ejido, Almería, Spain.
