# Supplementary material for: Strong-LAMP Assay Based on a Strongyloides spp.-Derived Partial Sequence in the 18S rRNA as Potential Biomarker for Strongyloidiasis Diagnosis in Human Urine Samples
Source: Dis Markers. 2020 May 31;2020:5265198. doi: 10.1155/2020/5265198 (PMC7281818; doi:10.1155/2020/5265198)
Supplement: Supplementary Materials — Table 1. Individual patients results from the Hospital Clínic de Barcelona, Barcelona, Spain. Table 2. Individual patients results from Hospital de Poniente, El Ejido, Almería, Spain. [file 5265198.f1.docx]

**Supplementary material.**

**Table 1**. Individual patients´ results from the Hospital Clínic de Barcelona, Barcelona, Spain

| ID | Origin | Laboratory Test | | |
| --- | --- | --- | --- | --- |
|  |  | IVD-ELISA (OD*) | Coproparasitological analysis | Strong-LAMP |
| 1 | Bolivia | 4.405 | Positive | Positive |
| 2 | Bolivia | 1.515 | Negative | Negative |
| 3 | Ecuador | 13.75 | Negative | Positive |
| 4 | Bolivia | 2.222 | Negative | Positive |
| 5 | Bolivia | 14 | Positive | Positive |
| 6 | Bolivia | 1.53 | Negative | Negative |
| 7 | Bolivia | 1.23 | Negative | Negative |
| 8 | Bolivia | 4 | Positive | Positive |
| 9 | Colombia | 2.0175 | Negative | Negative |
| 10 | Cuba | 1.705 | Negative | Negative |
| 11 | Spain | 7.24 | Negative | Negative |
| 12 | Spain | 2.192 | Negative | Negative |
| 13 | Ecuador | 0.005 | Positive | Positive |
| 14 | Portugal | 2.07 | Negative | Negative |
| 15 | Bolivia | 1.267 | Negative | Negative |
| 16 | Bolivia | 11.9 | Negative | Negative |

* Optical Density (OD) ≥1 were considered positive (according to the maker’s specifications).

**Table 2.** Individual patients´ results from Hospital de Poniente, El Ejido, Almería, Spain

| ID | Origin | Laboratory Test | | | |
| --- | --- | --- | --- | --- | --- |
|  |  | IVD-ELISA (OD*) | Ritchie  Technique | Coproparasitological analysis | Strong-LAMP |
| 17 | Gambia | 4.525 | Positive | Positive | Positive |
| 18 | Guinea Bissau | 4.105 | Positive | Positive | Positive |
| 19 | Guinea Bissau | 1.39 | Positive | Positive | Negative |
| 20 | Gambia | 1.975 | Negative | Negative | Negative |
| 21 | Mali | 0.48 | Positive | Negative | Positive |
| 22a | Nigeria | 3.22 | Positive | Positive | Positive |
| 23 | Gambia | 8.31 | Negative | Positive | Positive |
| 24 | Gambia | 0.39 | Positive | Negative | Positive |

* Optical Density (OD) ≥1 were considered positive (according to the maker’s specifications).

a. Patient HIV+ State A2.
